# Joint association of a healthy low-carbohydrate diet and frailty with the risk of incident Alzheimer's disease and vascular dementia: a prospective cohort study

**DOI:** 10.3389/fpubh.2026.1853648

**Published:** 2026-06-11

**Authors:** Zhongwei Zhang, Mengyun Hu, Zhiwei Teng, Yundi Mu, Ping Yin

**Affiliations:** 1 Department of Epidemiology and Biostatistics, School of Public Health, Tongji Medical College, Huazhong University of Science and Technology, Wuhan, China; 2 Department of Nursing, Union Hospital, Tongji Medical College, Huazhong University of Science and Technology, Wuhan, China; 3 Department of Nutrition and Food Hygiene, School of Public Health, Tongji Medical College, Huazhong University of Science and Technology, Wuhan, China

**Keywords:** Alzheimer's disease, frailty, healthy low-carbohydrate diet, joint association, vascular dementia

## Abstract

**Background:**

We aimed to investigate the associations between a healthy low-carbohydrate diet (HLCD) score, frailty and their combined effects with the risk of Alzheimer's disease (AD) and vascular dementia (VD).

**Methods:**

This cohort study analyzed 157,465 participants from the UK Biobank. The HLCD score was calculated based on the intake of total carbohydrates, vegetable proteins, and unsaturated fats, while frailty was assessed using a frailty index incorporating a wide range of biological systems and physical capacities, including sensory functions, mental health, and systemic comorbidities, which was developed and validated by the UK Biobank. Cox proportional hazards models and restricted cubic splines were employed for analysis.

**Results:**

Higher HLCD scores were significantly associated with a lower risk of AD (Q4 vs. Q1: HR = 0.66; 95% CI: 0.52–0.84) and VD (Q4 vs. Q1: HR = 0.54; 95% CI: 0.39–0.75). Frailty was identified as a risk factor for both dementia subtypes. Compared with the nonfrail group, the adjusted HRs for AD were 1.33 (1.08–1.65) for prefrail and 1.62 (1.33–1.98) for frail groups, while the adjusted HRs for VD were 1.61 (1.20–2.17) for prefrail and 2.46 (1.89–3.20) for frail groups. In the joint association analysis, adherence to a high-quality HLCD effectively mitigates the increased risk of AD attributed to frailty. Frail individuals with high HLCD scores exhibited a significantly lower risk of AD compared to those with low HLCD scores (HR = 0.63, 95% CI: 0.47–0.85). For VD, the protective benefit was more pronounced in the prefrailty stage (HR = 0.52, 0.35–0.79) than in the frail stage (HR = 0.83, 0.59–1.17).

**Conclusions:**

High adherence to the HLCD was associated with a lower risk of AD and VD, whereas frailty independently was associated with an increased the risk of AD and VD. Adherence to a high-quality HLCD effectively mitigates the increased risk associated with frailty. Promoting healthy low-carbohydrate diets may serve as a robust strategy to enhance cognitive resilience in older populations.

## 1 Background

As the global population continues to age, dementia has become a major public health challenge threatening the health of middle-aged and older adults. Dementia is a collective term for a group of neurodegenerative diseases that affect cognitive function; it is characterized by a significant decline in cognitive abilities, affecting memory, communication and the ability to carry out daily activities (1). Alzheimer's disease (AD) and vascular dementia (VD), as the main subtypes, not only severely impair patients' quality of life but also place a heavy economic burden on society and the healthcare system (2, 3). According to Alzheimer's Disease International (ADI), over 55 million people worldwide were living with dementia in 2020, a figure projected to reach 78 million by 2030 and 139 million by 2050 (4, 5). AD remains the most prevalent cause (6), accounting for 50%−75% of cases, followed by VD, which comprises 17%−30% of the dementia population (4). As there is currently no specific medication capable of curing dementia, identifying modifiable risk factors and developing early prevention strategies has become a central focus of geriatric medicine research.

Dietary patterns, as a modifiable lifestyle factor, play a significant role in the prevention of dementia (7–9). In recent years, low-carbohydrate diets (LCD) have attracted considerable attention due to their potential benefits in regulating glucose and lipid metabolism, reducing inflammatory responses and improving neurodegenerative conditions (10, 11). However, not all low-carbohydrate diets are beneficial to health; compared with diets that simply restrict carbohydrates, “healthy low-carbohydrate diets” (HLCD), which emphasize the intake of plant-based proteins and fats, are considered to offer better cardiometabolic and neuroprotective benefits (12–14). Previous studies have established three low-carbohydrate diet (LCD) scores: overall (OLCD), healthy (HLCD) and unhealthy (ULCD) (15). In short, macronutrients are categorized into high-quality and low-quality carbohydrates, animal and plant proteins, and saturated and unsaturated fats (16). The “Healthy Low-Carbohydrate Diet Score” is derived from the intake of low-quality carbohydrates, plant-based protein and unsaturated fats (17, 18). This scoring system employs a two-way scoring mechanism: protein and fat are scored positively (the higher the intake, the higher the score), whilst carbohydrate intake is scored negatively (the lower the intake, the higher the score). Unlike the OLCD, the HLCD emphasizes the quality of these macronutrients, with the aim of distinguishing healthier patterns within low-carbohydrate diets. Several studies have assessed the association between low-carbohydrate diets (LCD) and dementia (19–21), but the results have been inconsistent. For example, one prospective cohort study suggested that LCD reduces the risk of dementia (20), whereas another 9-year follow-up cohort study found a U-shaped association between LCD and dementia (21). At present, there remains a lack of evidence from large-scale population studies confirming the long-term prospective association between HLCD and different types of dementia (particularly AD and VD). The link between diet and dementia is mediated through complex biological pathways, including oxidative stress and metabolic disorders. For example, the development of insulin resistance and disrupted signaling pathways in the brains of AD patients (22, 23), as well as a reduction in the levels of the two main glucose transporters (GLUT1 and GLUT3) during the course of AD, are associated with the hyperphosphorylation of tau protein and the density of neurofibrillary tangles in the brain, which are characteristic features of AD (24). Furthermore, similar mechanisms such as oxidative stress and inflammatory responses are also present in the biological mechanisms underlying VD (25). Notably, cerebrovascular dysfunction characterized by endothelial impairment and blood-brain barrier disruption plays a central role in the pathogenesis of VD. HLCD may exert a potential neuroprotective effect by improving vascular endothelial function and reducing the severity of atherosclerosis (26).

At the same time, frailty—as a composite indicator reflecting a decline in an individual's physiological reserves and impairment of multiple systems—has been widely recognized as a strong predictor of dementia (27, 28). Frailty and poor dietary habits often co-occur and may accelerate cognitive decline through shared mechanisms such as oxidative stress and metabolic dysfunction (29, 30), however, there is currently a lack of evidence to suggest whether a high-quality dietary pattern (such as the HLCD) can effectively mitigate the increased dementia risk associated with frailty. Currently, clinical studies tend to examine diet and frailty as independent risk factors; however, in real life, an individual's health status is the result of a complex interplay of multiple factors, and there is limited evidence regarding the combined effects of HLCD and frailty on AD and VD. Furthermore, while the association between diet and frailty is established (31–33), it remains unclear whether the neuroprotective effects of HLCD are direct or partially mediated by the improvement of frailty status.

Therefore, in this study, we aim to investigate the long-term association between HLCD scores and the incidence of AD and VD through a large-scale population-based cohort study of UK adults, and to explore the dose-response relationship; secondly, we will further analyze the joint association between HLCD and frailty in relation to the risk of dementia. Finally, we will perform a mediation analysis to quantify the potential mediating role of frailty in the association between HLCD and dementia, thereby providing a more nuanced understanding of the dietary-cognitive axis. We hypothesized that higher HLCD scores would independently lower dementia risk while mitigating the adverse effects of frailty, which we further posited as a potential mediator in this relationship.

## 2 Materials and methods

### 2.1 Study design and setting

Over 500,000 people between the ages of 37 and 73 who were recruited from England, Scotland, and Wales between 2006 and 2010 make up the UK Biobank (UKB), a sizable prospective cohort (34). Each participant provided comprehensive data during the baseline visit. These data came from biological samples, physical measures, verbal interviews, and touch-screen questionnaires. All participants gave written informed consent prior to participation, and the study was approved by the ethics committee. Every procedure involving human participants complied with the institutional research committee's rules and the 1964 Helsinki Declaration's tenets.

Of the 501,931 participants in the UK Biobank, we included participants who had completed the online 24-h diet recall questionnaire at least once. Participants lacking 24-h dietary recall or frailty data were excluded (*n* = 294,459), accounting for more than half of the initial cohort. Participants were excluded if they met any of the following criteria: (1) withdrew from the study (*n* = 158); (2) had AD or VD at baseline (*n* = 3); (3) had missing information on covariates (*n* = 49,846). After the above exclusions, there were 157,465 participants in our final analysis (Supplementary Figure 1).

### 2.2 Healthy low-carbohydrate diet score

Dietary intake was measured using the Oxford WebQ, a web-based 24-h dietary recall questionnaire (https://biobank.ndph.ox.ac.uk/showcase/refer.cgi?id=118240), which collects information on the types and quantities of foods consumed, including beverages and daily nutrient intake (35, 36). During the final phase of UK Biobank recruitment (2009–2010), approximately 70,000 participants completed the Oxford WebQ survey in person at baseline assessment centers. Subsequently, the research team sent out four follow-up invitations in stages; the remaining participants completed the questionnaire remotely via personal computers at intervals of 3–4 months between February 2011 and June 2012. For participants who submitted dietary records on multiple occasions, the analysis utilized the average of their various indicators to reflect their long-term dietary habits and more accurately reduce the variability bias associated with single occasion reporting. In the UK Biobank, the “Estimated food nutrients yesterday” category provides estimates of nutrient intake based on participants' responses to the diet questionnaire (37). In this category, it specifically includes the estimated intake of total carbohydrates (Field ID: 26013), vegetable proteins (Field ID: 26006), monounsaturated fatty acids (Field ID: 26032), n-3 fatty acids (Field ID: 26015), n-6 fatty acids (Field ID: 26016), and energy (Field ID: 26002) from overall diet over the past 24 h. The intake of unsaturated fat is equal to the sum of monounsaturated fatty acids, n-3 fatty acids, and n-6 fatty acids. These nutrient quantities are directly available within the dataset, requiring no further calculation from specific food composition data (37). Given the data constraints of the UK Biobank, we developed a modified version of the original HLCD score. Specifically, our scoring system utilized total carbohydrate intake without differentiating by quality. To capture the “healthy” characteristics of the dietary pattern, our index emphasizes the replacement of carbohydrates with high-quality vegetable proteins and unsaturated fats.

Participants were categorized into 11 strata based on their energy intake percentages from total carbohydrates, vegetable proteins, and unsaturated fats. For vegetable proteins and unsaturated fats, scores were assigned in ascending order, ranging from 0 for the lowest stratum to 10 for the highest. In contrast, the scoring for total carbohydrate intake was reversed, with the lowest intake receiving the highest score. The HLCD score (total range: 0–30) was then derived by summing the individual scores from these three nutritional components (15, 17). The specific ranges for each of the 11 strata are detailed in Supplementary Table 1. Accordingly, elevated scores reflect reduced carbohydrate intake and increased protein and fat intake.

### 2.3 Frailty

The frailty index was the method used to quantify frailty at baseline, incorporating a wide range of biological systems and physical capacities, including sensory functions, mental health, and systemic comorbidities. A series of 49 self-reported items about health, illnesses, physical limits, and mental wellbeing traits were used to calculate the frailty index. This version of the frailty index has been previously developed and validated within the UKB cohort (Supplementary Table 2) (38). We computed the frailty index score for those with less than 10 missing items as the ratio of current deficits to all potential deficits, yielding a value between 0 and 1. Participants were classified as non-frail ( ≤ 0.12), prefrail (0.12–0.24), or frail (>0.24) based on this score (39, 40).

### 2.4 Outcome

Incident cases of dementia were identified by linking National Health Service records, which included hospital inpatient data and death registration records. Details about the linkage technique can be obtained at https://www.ukbiobank.ac.uk. Hospital admissions data were available until October 31, 2022, while mortality data were available until December 19, 2022. Follow-up was censored on these dates. AD and VD were diagnosed using the International Classification of Diseases, 10th revision (ICD-10) codes F00 and F01. The first instances of AD and VD outcomes were identified by mapping primary care data, hospital admissions data, death registry records, self-reported medical problems, and other sources (UKB data-field 132072). Follow-up duration was calculated from the attendance at the assessment center (Field 53) to the first diagnosis of AD or VD, death (UKB data-field 40000), or the last available date from the hospital inpatient data or primary care data. The first instance of AD or VD, death, or the censoring date, whichever occurred first, served as the follow-up endpoint. The date of each participant's most recent survey participation served as the censoring date.

### 2.5 Covariates

Covariates consisted of sociodemographic characteristics, lifestyle factors, and other potential confounding factors. These covariates included age, sex, ethnicity, Townsend deprivation index, educational level, physical activity level, particulate matter with diameter ≤ 2.5 μm (PM2.5), alcohol intake frequency, smoking status, Body Mass Index (BMI), diabetes and hypertension. Detailed covariate information is presented in Supplementary Table 3.

### 2.6 Statistical analysis

For quantitative variables, we displayed baseline features as mean (SD) or median Interquartile range (IQR); for categorical factors, we used *n* (%) by HLCD score (Q1, Q2, Q3, Q4). Analysis of Variance (ANOVA) tests for continuous variables and chi-squared tests for categorical variables were used to assess differences in attributes. The hazard ratios (HRs) and 95% confidence intervals (CIs) of HLCD score and frailty with AD or VD were evaluated using Cox proportional hazards models. Three Cox models were fitted: Model 1 was unadjusted; Model 2 was adjusted for age, sex, Townsend scores, and ethnicity; Model 3 was additionally adjusted for educational level, smoking status, alcohol intake, BMI, physical activity, PM2.5, hypertension, and diabetes on Model 2. In addition, the HLCD score was entered into the Cox models as a continuous variable (per 1-point increment) to complement the grouped analysis results. To examine the dose-response relationship between HLCD scores and the incidence of AD and VD, we utilized restricted cubic splines (RCS) with four knots at the 5th, 35th, 65th, and 95th percentiles. The *P*-value for non-linearity was calculated by testing the null hypothesis that the coefficients of the second and third splines are equal to zero. To evaluate the joint association of the HLCD score and frailty with the risk of incident AD and VD, we categorized participants into six mutually exclusive groups based on their HLCD and FI levels. Considering the sample size distribution, the HLCD score was dichotomized into “Low” (below the median) and “High” (at or above the median) levels, while frailty status was classified into three levels: nonfrail, prefrail, and frail. The group characterized by a Low HLCD score and frailty was designated as the reference group due to its hypothesized highest risk. To further investigate whether the observed associations between HLCD and dementia were mediated by frailty, we performed a formal mediation analysis using the Quasi-Bayesian Monte Carlo method via the mediation package in R. This approach allowed us to decompose the total effect into the average causal mediation effect (ACME) and the average direct effect (ADE). The proportion mediated was calculated to quantify the extent of the indirect effect of HLCD through frailty. CIs were estimated based on 1,000 simulations. All mediation models were adjusted for the same set of covariates used in the primary Cox proportional hazards models. We conducted four sensitivity analyses to evaluate the robustness of our main results. First, Stratified analyses were performed based on age, sex, BMI, hypertension, and diabetes to examine the association across subgroups to evaluate the consistency of the association between the HLCD score and AD and VD. Second, we further adjusted for total daily energy intake to ensure the robustness of the primary associations. Third, cases of AD and VD occurring within the first 5 years of follow-up were excluded to eliminate the potential effects of reverse causation.

All statistical analyses were carried out by SAS 9.4 (SAS Institute Inc., Cary, NC, USA) and R software (Version 4.4.2) (R Foundation for Statistical Computing, Vienna, Austria). All *P*-values were two-sided, and *P* < 0.05 was considered statistically significant.

## 3 Result

### 3.1 Baseline

The study included a total of 157,465 participants, with a mean follow-up period of 13.39 years (Table 1). During this period, we documented 558 incident cases of AD and 318 incident cases of VD, representing a cumulative incidence of 0.35 and 0.20%, respectively. As the HLCD score increased (from Q1 to Q4), the mean age of participants decreased slightly (Q1: 56.06 years vs. Q4: 55.48 years, *P* < 0.001). In the Q4 group, the proportion of women (56.5%) and those with a higher education level (16.5%) were significantly higher than in the Q1 group (women: 51.6%; higher education level: 13.7%; *P* < 0.001). In terms of lifestyle, a high HLCD score was associated with healthier behavioral patterns. The Q4 group had a lower mean BMI (26.76 kg/m^2^) and a significantly lower prevalence of hypertension (61.9%) compared with the Q1 group (64.7%; *P* < 0.001). Although there were statistically significant differences in alcohol consumption and smoking status between groups, the proportions of those who never drank alcohol or smoked did not show a clear linear concentration trend within the Q4 group. The distribution of frailty status differed significantly across the different HLCD groups, with the Q4 group exhibiting a higher proportion of nonfrail individuals (55.6%) and a lower proportion of prefrail and frail individuals (*P* < 0.001).

**Table 1:** Baseline characteristics of the study participants.

Characteristic	Overall *N* = 157,465	HLCD score				*p*-value^a^
		Q1 *N* = 39,367	Q2 *N* = 39,366	Q3 *N* = 39,366	Q4 *N* = 39,366	
Follow-up (years)	13.39 (0.85)	13.37 (0.85)	13.39 (0.84)	13.40 (0.85)	13.39 (0.85)	< 0.001
Age (years)	55.86 (7.97)	56.06 (8.07)	56.12 (7.95)	55.79 (7.98)	55.48 (7.86)	< 0.001
Townsend score	−1.62 (2.83)	−1.67 (2.84)	−1.70 (2.80)	−1.65 (2.81)	−1.47 (2.89)	< 0.001
Sex (female, %)	83,434 (53.0%)	20,312 (51.6%)	20,045 (50.9%)	20,846 (53.0%)	22,231 (56.5%)	< 0.001
Physical activity (%)						
Inactive	71,642 (45.5%)	17,763 (45.1%)	17,859 (45.4%)	18,231 (46.3%)	17,789 (45.2%)	0.002
Active	85,823 (54.5%)	21,604 (54.9%)	21,507 (54.6%)	21,135 (53.7%)	21,577 (54.8%)	
PM2.5 (μg/m^3^)	9.91 (1.03)	9.89 (1.00)	9.89 (1.02)	9.90 (1.03)	9.95 (1.05)	< 0.001
Education						
High	23,969 (15.2%)	5,385 (13.7%)	5,942 (15.1%)	6,131 (15.6%)	6,511 (16.5%)	< 0.001
Intermediate	122,182 (77.6%)	30,558 (77.6%)	30,542 (77.6%)	30,591 (77.7%)	30,491 (77.5%)	
Low	11,314 (7.2%)	3,424 (8.7%)	2,882 (7.3%)	2,644 (6.7%)	2,364 (6.0%)	
Ethnicity						
White	150,748 (95.7%)	37,373 (94.9%)	37,932 (96.4%)	37,879 (96.2%)	37,564 (95.4%)	< 0.001
Others	6,717 (4.3%)	1,994 (5.1%)	1,434 (3.6%)	1,487 (3.8%)	1,802 (4.6%)	
Body mass index (kg/m^2^)	26.86 (4.56)	26.96 (4.48)	26.87 (4.45)	26.86 (4.62)	26.77 (4.69)	< 0.001
Alcohol intake (%)						
< 3 times/week	70,348 (44.7%)	19,201 (48.8%)	17,085 (43.4%)	16,743 (42.5%)	17,319 (44.0%)	< 0.001
≥3 times/week	77,827 (49.4%)	17,110 (43.5%)	20,139 (51.2%)	20,612 (52.4%)	19,966 (50.7%)	
Never	9,290 (5.9%)	3,056 (7.8%)	2,142 (5.4%)	2,011 (5.1%)	2,081 (5.3%)	
Smoking status (%)						
Current	12,048 (7.7%)	3,014 (7.7%)	2,869 (7.3%)	3,037 (7.7%)	3,128 (7.9%)	< 0.001
Never	89,010 (56.5%)	22,957 (58.3%)	22,620 (57.5%)	22,046 (56.0%)	21,387 (54.3%)	
Previous	56,407 (35.8%)	13,396 (34.0%)	13,877 (35.3%)	14,283 (36.3%)	14,851 (37.7%)	
Hypertension (%)	100,502 (63.8%)	25,468 (64.7%)	25,589 (65.0%)	25,074 (63.7%)	24,371 (61.9%)	< 0.001
Diabetes (%)	21,004 (13.3%)	4,884 (12.4%)	5,218 (13.3%)	5,331 (13.5%)	5,571 (14.2%)	< 0.001
Total energy intake (kcal/day)	2,076.64 (603.01)	1,972.85 (589.60)	2,074.96 (579.55)	2,125.95 (596.79)	2,132.82 (631.24)	< 0.001
Frailty index						
Nonfrail	87,643 (55.7%)	21,337 (54.2%)	22,184 (56.4%)	22,239 (56.5%)	21,883 (55.6%)	< 0.001
Prefrail	35,944 (22.8%)	9,160 (23.3%)	8,957 (22.8%)	8,823 (22.4%)	9,004 (22.9%)	
Frail	33,878 (21.5%)	8,870 (22.5%)	8,225 (20.9%)	8,304 (21.1%)	8,479 (21.5%)	

^a^One-way analysis of means; Pearson's Chi-squared test.

HLCD, healthy low-carbohydrate diet.

### 3.2 Independent associations of HLCD scores with the risk of Alzheimer's disease and vascular dementia

**Figure 1:**
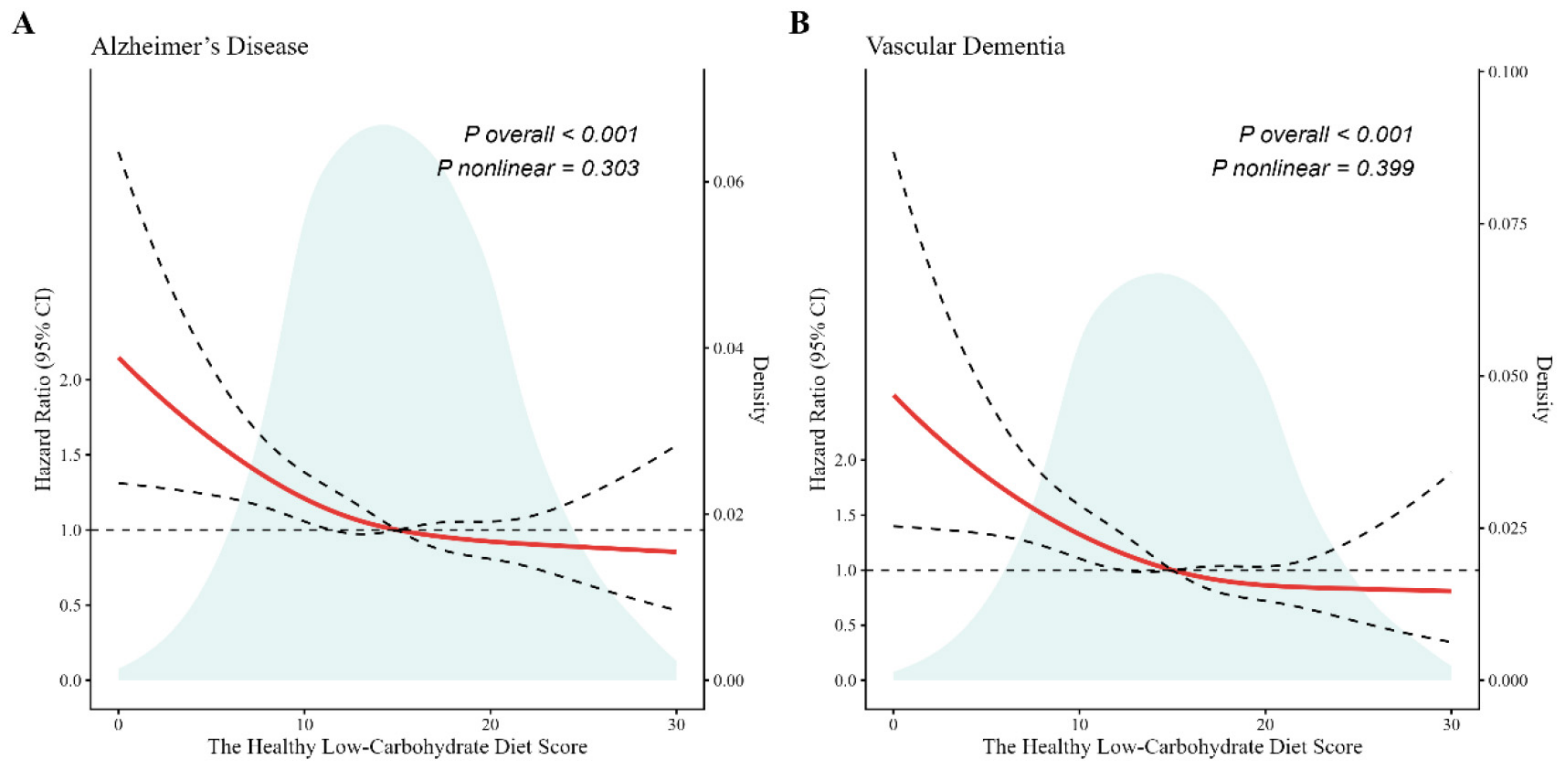
Restricted cubic spline regression models of the associations between the HLCD score and risks of AD and VD. **(A)** AD; **(B)** VD. The model was adjusted for the same confounding variables as Model 3 presented above. HR, hazard ratio; CI, confidence interval.

Independent association analysis revealed a significant inverse correlation between HLCD scores and the risk of both AD and VD (Table 2). As HLCD quartiles increased, the risk of developing both types of dementia showed a marked downward gradient. In the fully adjusted model (Model 3), compared with the lowest HLCD score group (Q1), the highest group (Q4) had a 34% lower risk of developing AD (HR = 0.66; 95% CI: 0.52–0.84; *P* < 0.001). Furthermore, analysis of the continuous variable showed that for every 1-point increase in the HLCD score, the risk of AD was independently reduced by 3% (HR = 0.97; 95% CI: 0.95–0.99; *P* < 0.001). For VD, the results of Model 3 showed a 46% reduction in risk in the Q4 group (HR = 0.54; 95% CI: 0.39–0.75; *P* < 0.001). For every 1-point increase in the HLCD score, the risk of VD was reduced by 4% (HR = 0.96; 95% CI: 0.94–0.98; *P* < 0.001). The results of the RCS analysis indicate that there is a significant linear dose-response relationship between the HLCD score and the risk of AD and VD (AD: *P*_overall_ < 0.001; *P*_nonlinear_ = 0.303; VD: *P*_overall_ < 0.001, *P*_nonlinear_ = 0.339; Figure 1). As the HLCD score increased, the risk of developing both types of dementia showed a continuous downward trend.

**Table 2:** Associations between the healthy low-carbohydrate diet score and risks of Alzheimer's disease and vascular dementia.

Outcome	Variable	Events/*N*	Model 1	*p*-value	Model 2	*p*-value	Model 3	*p*-value
			HR (95% CI)		HR (95% CI)		HR (95% CI)	
AD	Q1	183/39,367	1.00 (Ref)	—	1.00 (Ref)	—	1.00 (Ref)	—
	Q2	145/39,366	0.78 (0.63–0.97)	0.029	0.79 (0.63–0.98)	0.033	0.80 (0.65–1.00)	0.050
	Q3	124/39,366	0.67 (0.53–0.84)	< 0.001	0.71 (0.56–0.89)	0.003	0.72 (0.57–0.91)	0.005
	Q4	106/39,366	0.57 (0.45–0.73)	< 0.001	0.64 (0.51–0.82)	< 0.001	0.66 (0.52–0.84)	< 0.001
	HLCD (per 1-unit)		0.96 (0.95–0.98)	< 0.001	0.97 (0.95–0.98)	< 0.001	0.97 (0.95–0.99)	< 0.001
VD	Q1	115/39,367	1.00 (Ref)	—	1.00 (Ref)	—	1.00 (Ref)	—
	Q2	80/39,366	0.68 (0.51–0.90)	0.007	0.67 (0.50–0.89)	0.006	0.68 (0.51–0.91)	0.010
	Q3	69/39,366	0.59 (0.44–0.80)	< 0.001	0.63 (0.47–0.85)	0.002	0.65 (0.48–0.87)	0.004
	Q4	54/39,366	0.46 (0.33–0.64)	< 0.001	0.53 (0.38–0.73)	< 0.001	0.54 (0.39–0.75)	< 0.001
	HLCD (per 1-unit)		0.95 (0.93–0.97)	< 0.001	0.95 (0.93–0.97)	< 0.001	0.96 (0.94–0.98)	< 0.001

HLCD, healthy low-carbohydrate diet; AD, Alzheimer's disease; VD, vascular dementia; HR, hazard ratio; CI, confidence interval.

Model 1: unadjusted. Model 2: adjusted for age, sex, Townsend scores and ethnicity. Model 3: additionally adjusted for education, PM2.5, smoking status, alcohol intake, BMI, physical activity, hypertension and diabetes on Model 2.

### 3.3 Associations between frailty and risks of AD and VD

Independent association analysis revealed that frailty was positively associated with the risk of both AD and VD (Table 3). As the degree of frailty increased, the risk of dementia showed a significant stepwise increase. In the fully adjusted model (Model 3), compared with the nonfrail group, the risk of AD was 33% higher in the prefrail group (HR = 1.33; 95% CI: 1.08–1.65), whilst the risk in the frail group increased significantly by 62% (HR = 1.62; 95% CI: 1.33–1.98). The impact of frailty on the risk of VD was even more pronounced. After adjusting for demographic and lifestyle factors (Model 3), the risk of VD in the frail group was 1.46 times higher than in the nonfrail group (HR = 2.46; 95% CI: 1.89–3.20). Even in the prefrail stage, the risk of VD was independently increased by 61% (HR = 1.61; 95% CI: 1.20–2.17).

**Table 3:** Associations between the frailty and risks of Alzheimer's disease and vascular dementia.

Outcome	Variable	Events/*N*	Model 1	*p*-value	Model 2	*p*-value	Model 3	*p*-value
			HR (95% CI)		HR (95% CI)		HR (95% CI)	
AD	Nonfrail	232/87,643	1.00 (Ref)		1.00 (Ref)		1.00 (Ref)	
	Prefrail	143/35,944	1.53 (1.24–1.89)	< 0.001	1.35 (1.09–1.66)	0.005	1.33 (1.08–1.65)	0.008
	Frail	183/33,878	2.12 (1.75–2.58)	< 0.001	1.61 (1.33–1.96)	< 0.001	1.62 (1.33–1.98)	< 0.001
VD	Nonfrail	101/87,643	1.00 (Ref)		1.00 (Ref)		1.00 (Ref)	
	Prefrail	82/35,944	2.03 (1.52–2.72)	< 0.001	1.80 (1.34–2.41)	< 0.001	1.61 (1.20–2.17)	0.002
	Frail	135/33,878	3.61 (2.79–4.67)	< 0.001	2.67 (2.06–3.47)	< 0.001	2.46 (1.89–3.20)	< 0.001

AD, Alzheimer's disease; VD, vascular dementia; HR, hazard ratio; CI, confidence interval.

Model 1: unadjusted. Model 2: adjusted for age, sex, Townsend scores and ethnicity. Model 3: additionally adjusted for education, PM2.5, smoking status, alcohol intake, BMI, physical activity, hypertension and diabetes on Model 2.

### 3.4 Joint effects of HLCD scores and frailty status on the risk of AD and VD

**Figure 2:**
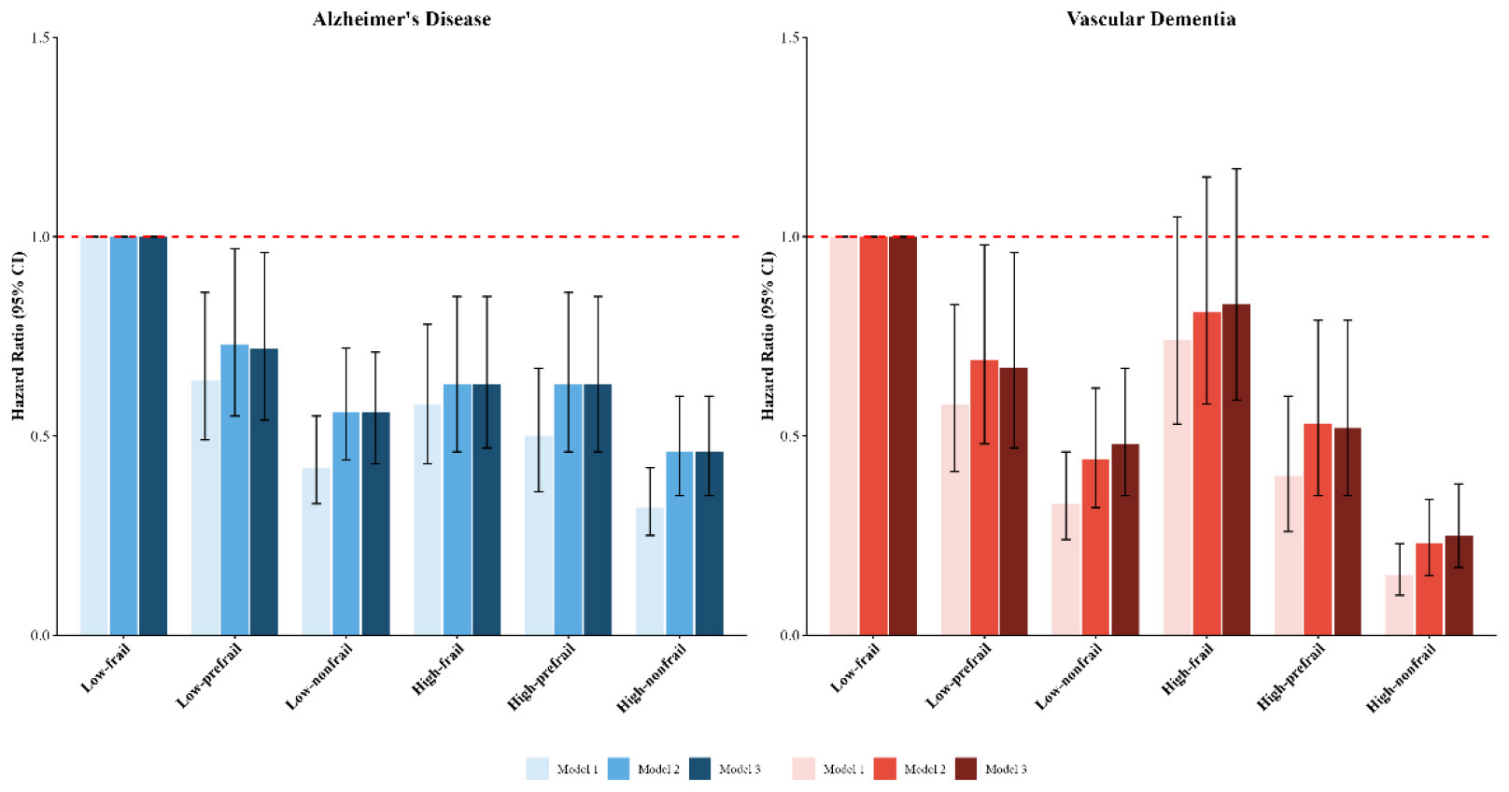
Joint association of HLCD score and frailty with the risk of Alzheimer's disease and vascular dementia. HR, hazard ratio; CI, confidence interval. Model 1: unadjusted. Model 2: adjusted for age, sex, Townsend scores and ethnicity. Model 3: additionally adjusted for education, PM2.5, smoking status, alcohol intake, BMI, physical activity, hypertension and diabetes on Model 2.

Joint association analysis revealed a significant cumulative effect of HLCD scores and frailty on the risk of dementia (Figure 2). Using the “low HLCD and frail” cohort as the reference group, the risk of AD and VD showed a clear downward gradient as dietary quality improved, and frailty decreased. For AD, high adherence to the HLCD demonstrated protective benefits across all frailty levels. Notably, even among frail individuals, high HLCD adherence was still associated with a significant reduction in risk (HR = 0.63, 95% CI: 0.47–0.85). In the ideal group of “high HLCD adherence and nonfrail” individuals, the risk of AD was reduced by 54% (HR = 0.46, 95% CI: 0.35–0.60). In contrast, the protective effect of the combined intervention against VD was more pronounced in the prefrail stage. Among those with high HLCD and prefrail status, the risk of VD was reduced by 48% (HR = 0.52, 95% CI: 0.35–0.79); however, among those already in a frail state, the protective effect of high HLCD was not statistically significant (HR = 0.83, 95% CI: 0.59–1.17).

### 3.5 Mediation analysis of frailty

To further elucidate the potential biological pathways underlying the observed associations, we performed a formal mediation analysis to quantify the extent to which the protective effect of HLCD was mediated by frailty status. The results indicated that frailty significantly mediated the association between HLCD and the risk of both dementia subtypes. Specifically, for AD, the mediation effect was statistically significant (*P* < 0.001), with frailty accounting for 0.65% (95% CI: 0.19%−1.74%) of the total association. Similarly, for VD, the mediation effect was found to be statistically significant (*P* < 0.001), with a slightly higher mediated proportion of 0.94% (95% CI: 0.29%−2.21%). Full results of the mediation analysis are presented in Supplementary Table 4.

### 3.6 Subgroup analysis and sensitivity analysis

In subgroup analysis, the inverse association between the HLCD score and the risk of AD and VD was consistent across all subgroups (including age, sex, BMI, and baseline hypertension and diabetes status; Supplementary Figure 2). No significant interactions were observed between the HLCD score and these subgroup variables (all *P*_interaction_ > 0.05).

Multiple sensitivity analyses further confirmed the robustness of the associations between the HLCD score, frailty status and the risk of dementia (see Supplementary Tables 5–7). First, after further adjustment for total energy intake, the protective effect of HLCD against dementia remained significant, and its combined association pattern with frailty remained unchanged. Second, to rule out the confounding effect of reverse causality, we conducted a 5-year lag-time analysis. After excluding cases that developed the condition within the first 5 years of follow-up, the synergistic protective effect of a high HLCD score and a nonfrail status was highly consistent with the results of the main analysis (HR for AD = 0.50, 95% CI: 0.38–0.65; HR for VD = 0.28, 95% CI: 0.18–0.44).

## 4 Discussion

This study, utilizing large-scale prospective data from the UK Biobank, found that adherence to a HLCD was significantly associated with a reduced risk of AD and VD. At the same time, frailty was significantly and positively associated with the outcomes. A key finding is that there is a significant synergistic effect between the HLCD score and frailty on the risk of dementia: even among frail individuals, a high-quality dietary pattern can effectively mitigate the risk of dementia associated with frailty.

In recent years, numerous studies have examined the link between healthy diets and dementia, such as the Mediterranean diet (41, 42), the ketogenic diet (11, 43), the Mediterranean-DASH Intervention for Neurodegenerative Delay (MIND) diet (42, 44); most dietary patterns suggest that LCD may help reduce the risk of dementia. Some studies have also suggested a U-shaped relationship between carbohydrate intake and the risk of dementia (21). Previous studies have highlighted the importance of the quality and sources of macronutrients when promoting LCD (45). Therefore, compared with OLCD and ULCD, HLCD may offer superior benefits in reducing the risk of developing dementia.

In the pathogenesis of AD, insulin resistance in the brain (22, 46) and inflammatory responses are considered key drivers. The HLCD significantly improves systemic and central insulin sensitivity by reducing the glycemic load of the diet (47). Optimizing this metabolic state helps the insulin-degrading enzyme (IDE) to clear β-amyloid (Aβ) from the brain more effectively (48, 49), thereby alleviating the neurotoxicity caused by protein deposits. Furthermore, the plant-based components of HLCD regulate the activity of microglia, reduce levels of pro-inflammatory cytokines, and thereby inhibit at the source the chronic inflammatory processes that may lead to neuronal apoptosis (50, 51).

The protective effect of HLCD on VD is primarily attributed to its role in maintaining vascular integrity. The high proportion of unsaturated fatty acids [such as docosahexaenoic acid (DHA)] in this dietary pattern not only modulates vascular risk factors but also reduces neuroinflammation and oxidative damage that may lead to neurological dysfunction (52). The aforementioned mechanisms may be effective in the prevention and treatment of VD (53). At the same time, HLCD effectively regulates blood pressure, blood lipid levels and blood glucose levels, thereby reducing the risk of atherosclerosis and microthrombus formation (54, 55). As the development of vascular disease is closely associated with long-term small-vessel pathology (56), this comprehensive management of vascular risk factors may account for the unique advantages of HLCD in the prevention of VD. Furthermore, the lack of a significant non-linear association between the HLCD and AD and VD may suggest that the reduction in the risk of dementia is proportional to improvements in the quality of the HLCD, and that no clear threshold effect has been observed within the score range covered by the study.

Whilst examining dietary associations, this study further established that frailty was an independent predictor of the onset of AD and VD. Studies examining the association between frailty and dementia are not uncommon in the literature (57, 58). Frailty reflects a decline in an individual's multisystemic physiological reserves; the underlying systemic low-grade inflammation is closely linked to the loss of cognitive reserves in the brain and the acceleration of neurodegenerative diseases (27, 58). Consequently, using the frailty index as a tool for assessing the risk of dementia in screening the older population is of significant clinical value.

This study revealed the combined effect of HLCD and frailty on the risk of dementia. Adherence to an HLCD may mitigate the role of frailty in driving the risk of dementia. This suggests that, although frailty is a strong predictor of dementia, the protective effects of HLCD characterized by plant-based proteins and unsaturated fats can partially mitigate the risk of AD and VD specific to frail individuals. Our study identified nuanced differences in the dietary mitigating patterns between AD and VD. While high HLCD adherence provided consistent protection against AD across the entire frailty spectrum, its efficacy against VD appeared to be stage-dependent, peaking during the prefrail phase. This may be due to severe frailty and cerebrovascular damage undermining the protective effects of nutritional optimization, or because there were fewer patients with VD than with AD in the subgroup, resulting in insufficient statistical power. In summary, dietary interventions may still offer significant neuroprotective benefits for individuals who are already frail or prefrail.

Finally, our mediation analysis found that frailty significantly mediated the association between HLCD and both AD and VD. However, the proportion mediated by frailty was relatively modest (less than 1% for both subtypes). This suggests that while HLCD contributes to maintaining physiological robustness—which in turn buffers neurodegeneration—the vast majority of its neuroprotective benefits likely stem from the direct pathways mentioned above.

Drawing on the large-scale prospective cohort of the UK Biobank, we systematically investigated the association between HLCD and subtypes of dementia using over a decade of follow-up data, thereby significantly enhanced the reliability and generalizability of our findings. Second, in developing the HLCD score, this study made a clear distinction between different sources of nutrients, emphasizing the neuroprotective benefits of plant-based protein and unsaturated fats, rather than solely restricting total carbohydrate intake; this is more in line with the principles of modern precision nutrition interventions. Our findings emphasize a quality-based substitution strategy rather than mere carbohydrate restriction. To achieve a high HLCD score in practice, individuals should prioritize replacing a portion of total carbohydrates with nutrient-dense plant alternatives, such as legumes, nuts, and vegetable oils. This “plant-forward” approach translates our findings into actionable lifestyle recommendations by focusing on the source and balance of macronutrients to mitigate dementia risk. Furthermore, this study is the first to integrate dietary patterns and frailty into a unified research framework; through joint association analysis, it has revealed the “mitigating effect” of diet on high-risk populations, thereby providing new evidence of significant clinical value for the management of cognitive health in middle-aged and older adults.

Despite the rigorous study design, several inherent limitations remain. First, dietary data were derived from self-reported 24-h dietary recalls; although multiple measurements were averaged to reduce error, recall bias could not be entirely avoided, and the baseline assessment may not fully capture changes in dietary habits during the follow-up period. Second, as an observational study, even though we extensively adjusted for covariates such as demographic, lifestyle and environmental health factors, and conducted multiple sensitivity analyses to address potential reverse causation, unmeasured residual confounding factors may still influence the associations. Finally, participants in the UK Biobank exhibit a certain degree of the “healthy volunteer effect,” and their incidence rates and health awareness may differ from those of the general population; therefore, caution is required when extrapolating the findings to different ethnic groups or the very old populations.

## 5 Conclusion

This prospective study showed that high adherence to the HLCD was associated with a lower risk of AD and VD, whereas frailty independently was associated with an increased the risk of AD and VD. Adherence to a high-quality HLCD effectively mitigates the increased risk associated with frailty. The protective effects of the HLCD appear to be primarily direct, though partially explained by the mitigation of frailty.

## Data Availability

Publicly available datasets were analyzed in this study. This data can be found here: the UK Biobank data analyzed in this study is subject to the following licenses/restrictions: UK Biobank data are available to all researchers for health-related research and public interest through registration on the UK Biobank (www.ukbiobank.ac.uk). This research has been conducted using the UK Biobank Resource under Application Number 162275. Requests to access these datasets should be directed to Zhongwei Zhang, zhongweizzzz@163.com.
