# Supplementary material for: Joint association of a healthy low-carbohydrate diet and frailty with the risk of incident Alzheimer's disease and vascular dementia: a prospective cohort study
Source: Front Public Health. 2026 Jun 11;14:1853648. doi: 10.3389/fpubh.2026.1853648 (PMC13294439; doi:10.3389/fpubh.2026.1853648)
Supplement: Supplementary file 1 [file Data_Sheet_1.docx]

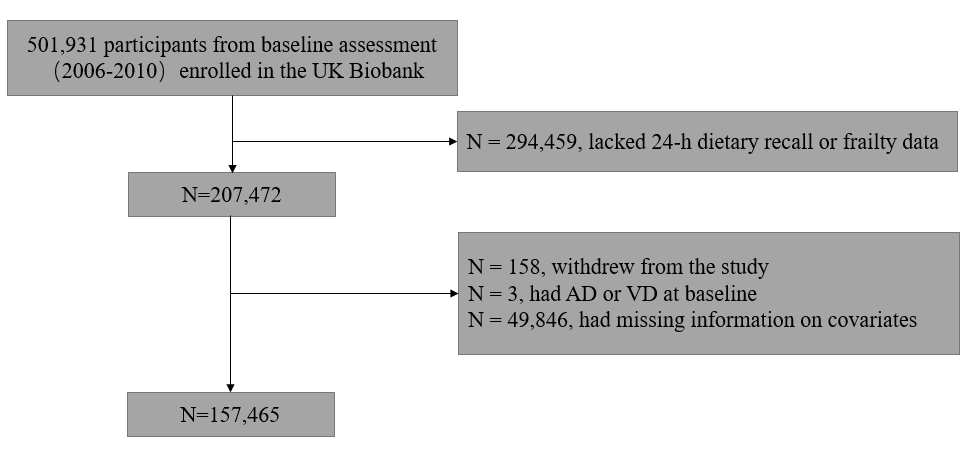


Supplementary Figure 1 The flowchart of participant selection


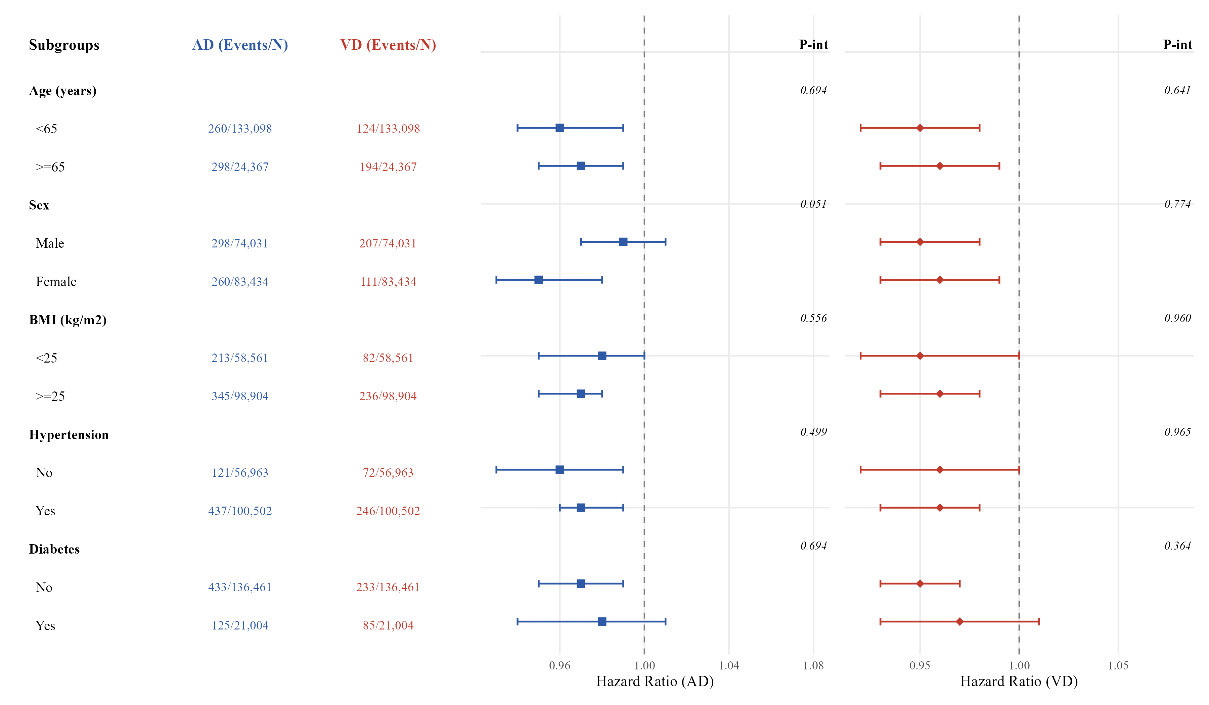


Supplementary Figure 2 Subgroup analyses of associations between the Healthy Low-Carbohydrate Diet score and risk of Alzheimer’s Disease and Vascular Dementia

Supplementary Table 1 Criteria for determining the healthy low-carbohydrate diet (HLCD) score.

| **Points** | **Total Carbohydrate Intake (%)** | **Vegetable Protein Intake (%)** | **Unsaturated Fat Intake (%)** |
| --- | --- | --- | --- |
| 0 | > 60.2 | < 3.8 | < 11.7 |
| 1 | 56.8-60.2 | 3.8-4.3 | 11.7-13.3 |
| 2 | 54.4-56.8 | 4.3-4.6 | 13.3-14.5 |
| 3 | 52.5-54.4 | 4.6-4.9 | 14.5-15.4 |
| 4 | 50.7-52.5 | 4.9-5.2 | 15.4-16.3 |
| 5 | 49.0-50.7 | 5.2-5.5 | 16.3-17.2 |
| 6 | 47.1-49.0 | 5.5-5.8 | 17.2-18.1 |
| 7 | 45.1-47.1 | 5.8-6.2 | 18.1-19.2 |
| 8 | 42.5-45.1 | 6.2-6.7 | 19.2-20.5 |
| 9 | 38.7-42,5 | 6.7-7.5 | 20.5-22.6 |
| 10 | < 38.7 | > 7.5 | > 22.6 |

Supplementary Table 2 Items from the baseline UKB assessment used to compose the frailty index

| Type of deficit | Item | Trait | UKB Data-Field | Coding in FI item |
| --- | --- | --- | --- | --- |
| Sensory | 1 | Glaucoma | 2227 | \| Yes = 1 No = 0 Missing if "prefer not to answer" for data-field 6148. \| \| --- \| |
|  |  |  | 6148 |  |
|  |  |  | 20002 |  |
|  | 2 | Cataracts | 6148 | \| Yes = 1 No = 0 Missing if "prefer not to answer" for data-field 6148. \| \| --- \| |
|  |  |  | 2227 |  |
|  |  |  | 20002 |  |
|  | 3 | Hearing difficulty | 2247 | \| Yes and completely deaf = 1 No = 0 \| \| --- \| |
| Cranial | 4 | Migraine | 2473 | \| Yes = 1 No = 0 Missing if "prefer not to answer" for data-field 2473 \| \| --- \| |
|  |  |  | 20002 |  |
|  | 5 | Dental problems | 6149 | \| None = 0 Any = 1 \| \| --- \| |
| Mental wellbeing | 6 | Self-rated health | 2178 | \| Excellent = 0 Good = 0.25 Fair = 0.5 Poor = 1 \| \| --- \| |
|  | 7 | Fatigue: frequency of tiredness / lethargy in last two weeks | 2080 | \| Not at all = 0 Several days = 0.25 More than half = 0.5 Nearly every day = 1 \| \| --- \| |
|  | 8 | Sleep: experience of sleeplessness/insomnia | 1200 | \| Never/rarely = 0 Sometimes = 0.5 Usually = 1 \| \| --- \| |
|  | 9 | Depressed feelings: frequency in last two weeks | 2050 | \| Not at all = 0 Several days = 0.5 More than half = 0.75 Nearly every day = 1 \| \| --- \| |
|  | 10 | Self-described nervous personality | 1970 | \| Yes = 1 No = 0 \| \| --- \| |
|  | 11 | Severe anxiety/ panic attacks | 2473 | \| Yes = 1 No = 0 Missing if "prefer not to answer" for data-field 2473. \| \| --- \| |
|  |  |  | 20002 |  |
|  | 12 | Common to feel loneliness | 2020 | \| Yes = 1 No = 0 \| \| --- \| |
|  | 13 | Sense of misery (ever/never) | 1930 | \| Yes = 1 No = 0 \| \| --- \| |
| Infirmity | 14 | Infirmity: long-standing illness or disability | 2188 | \| Yes = 1 No = 0 \| \| --- \| |
|  | 15 | Falls in last year | 2296 | \| No falls = 0 Only one fall = 0.5 More than one fall = 1 \| \| --- \| |
|  | 16 | Fractures/broken bones in last five years | 2463 | \| Yes = 1 No = 0 \| \| --- \| |
| Cardiometabolic | 17 | Diabetes | 2443 | \| Yes = 1 No = 0  Missing if "prefer not to answer" for data-field 2443. \| \| --- \| |
|  |  |  | 20002 |  |
|  | 18 | Myocardial infarction | 6150 | \| Yes = 1 No = 0 Missing if "prefer not to answer" for data-field 6150. \| \| --- \| |
|  |  |  | 20002 |  |
|  | 19 | Angina | 6150 | \| Yes = 1 No = 0 Missing if "prefer not to answer" for data-field 6150. \| \| --- \| |
|  |  |  | 20002 |  |
|  | 20 | Stroke | 6150 | \| Yes = 1 No = 0 Missing if "prefer not to answer" for data-field 6150. \| \| --- \| |
|  |  |  | 20002 |  |
|  | 21 | High blood pressure | 6150 | \| Yes = 1 No = 0 Missing if "prefer not to answer" for data-field 6150. \| \| --- \| |
|  |  |  | 20002 |  |
|  | 22 | Hypothyroidism | 2473 | \| Yes = 1 No = 0 Missing if "prefer not to answer" for data-field 2473. \| \| --- \| |
|  |  |  | 20002 |  |
|  | 23 | Deep-vein thrombosis | 6152 | \| Yes = 1 No = 0 Missing if "prefer not to answer" for data-field 6152. \| \| --- \| |
|  |  |  | 20002 |  |
|  | 24 | High cholesterol | 6153 | \| Yes = 1 No = 0 Missing if "prefer not to answer" for data-field 6153 or 6177. \| \| --- \| |
|  |  |  | 6177 |  |
| Respiratory | 25 | Breathing: wheeze in last year | 2316 | \| Yes = 1 No = 0 \| \| --- \| |
|  | 26 | Pneumonia | 2473 | \| Yes = 1 No = 0 Missing if "prefer not to answer" for data-field 2473. \| \| --- \| |
|  |  |  | 20002 |  |
|  | 27 | Chronic bronchitis/emphysema | 6152 | \| Yes = 1 No = 0 Missing if "prefer not to answer" for data-field 6152. \| \| --- \| |
|  |  |  | 20002 |  |
|  | 28 | Asthma | 6152 | \| Yes = 1 No = 0 Missing if "prefer not to answer" for data-field 6152. \| \| --- \| |
|  |  |  | 20002 |  |
| Musculoskeletal | 29 | Rheumatoid arthritis | 2473 | \| Yes = 1 No = 0 Missing if "prefer not to answer" for data-field 2473. \| \| --- \| |
|  |  |  | 20002 |  |
|  | 30 | Osteoarthritis | 2473 | \| Yes = 1 No = 0 Missing if "prefer not to answer" for data-field 2473. \| \| --- \| |
|  |  |  | 20002 |  |
|  | 31 | Gout | 2473 | \| Yes = 1 No = 0 Missing if "prefer not to answer" for data-field 2473. \| \| --- \| |
|  |  |  | 20002 |  |
|  | 32 | Osteoporosis | 2473 | \| Yes = 1 No = 0 Missing if "prefer not to answer" for data-field 2473. \| \| --- \| |
|  |  |  | 20002 |  |
| Immunological | 33 | Hayfever, allergic rhinitis or eczema | 6152 | \| Yes = 1 No = 0 Missing if "prefer not to answer" for data-field 6152. \| \| --- \| |
|  |  |  | 20002 |  |
|  | 34 | Psoriasis | 2473 | \| Yes = 1 No = 0 Missing if "prefer not to answer" for data-field 2473. \| \| --- \| |
|  |  |  | 20002 |  |
| Cancer | 35 | Any cancer diagnosis | 2453 | \| Yes = 1 No = 0 Missing if "prefer not to answer" for data-field 2453. \| \| --- \| |
|  |  |  | 134 |  |
|  | 36 | Multiple cancers diagnosed (number reported) | 134 | \| Range from 0 to 6; No cancer or single cancer = 0 Multiple cancers = 1 \| \| --- \| |
| Pain | 37 | Chest pain | 2335 | \| Yes = 1 No = 0 \| \| --- \| |
|  | 38 | Head and/or neck pain | 6159 | \| Yes (combining responses to pain in head and neck/shoulders) = 1 No = 0 \| \| --- \| |
|  | 39 | Back pain | 6159 | Yes = 1 No = 0 |
|  | 40 | Stomach/abdominal pain | 6159 | Yes = 1 No = 0 |
|  | 41 | Hip pain | 6159 | Yes = 1 No = 0 |
|  | 42 | Knee pain | 6159 | Yes = 1 No = 0 |
|  | 43 | Whole-body pain | 6159 | Yes = 1 No = 0 |
|  | 44 | Facial pain | 6159 | Yes = 1 No = 0 |
|  | 45 | Sciatica | 2473 | \| Yes = 1 No = 0 Missing if "prefer not to answer" for data-field 2473. \| \| --- \| |
|  |  |  | 20002 |  |
| Gastrointestinal | 46 | Gastric reflux | 2473 | Yes = 1 No = 0 Missing if "prefer not to answer" for data-field 2473. |
|  |  |  | 20002 |  |
|  | 47 | Hiatus hernia | 2473 | Yes = 1 No = 0 Missing if "prefer not to answer" for data-field 2473. |
|  |  |  | 20002 |  |
|  | 48 | Gall stones | 2473 | Yes = 1 No = 0 Missing if "prefer not to answer" for data-field 2473. |
|  |  |  | 20002 |  |
|  | 49 | Diverticulitis | 2473 | Yes = 1 No = 0 Missing if "prefer not to answer" for data-field 2473. |
|  |  |  | 20002 |  |

Supplementary Table 3 Covariate information for UK Biobank.

| UK Biobank | |
| --- | --- |
| covariate | details |
| Age | continuous |
| Sex | male or female |
| Ethnicity | white or non-white |
| Townsend deprivation index | continuous, with high values indicating high levels of deprivation |
| Educational level | high [college/university degree or above], intermediate [advanced/advanced subsidiary levels, ordinary levels, general certificate of secondary education, certificate of secondary education, national vocational qualification or higher national diploma, or equivalent, and other professional qualifications] and low [none of the above] |
| Particulate matter with diameter≤2.5μm (PM2.5) | continuous |
| Physical activity level | meeting or not meeting the physical activity guideline of 150 min of moderate activity or 75 min of vigorous activity per week |
| Alcohol intake frequency | never, less than 3 times/week, and ≥3 times/week |
| Smoking status | never, past, or current |
| Body mass index | continuous |
| High glycemia/Diabetes | fasting glucose ≥5.6 mmol/L or self-reported diabetes |
| High blood pressure/Hypertension | systolic blood pressure ≥130 mmHg and/or diastolic blood pressure ≥85 mmHg or self-reported hypertension |
| Total energy intake (kcal/day) | continuous |

Supplementary Table 4 Mediation analysis of the association between HLCD scores and dementia risk mediated by frailty

| Outcome | Effects | Estimate (95%CI) | P |
| --- | --- | --- | --- |
| AD | ACME (Average) | -1.06e-06 (-1.91e-06, -2.84e-07) | <0.001 |
|  | ADE (Average) | -1.59e-04 (-2.81e-04, -5.66e-05) | <0.001 |
|  | Total Effect | -1.60e-04 (-2.82e-04, -5.74e-05) | <0.001 |
|  | Prop. Mediated | 0.65% (0.19%, 1.74%) | <0.001 |
| VD | ACME (Average) | -1.50e-06 (-2.75e-06, -5.86e-07) | <0.001 |
|  | ADE (Average) | -1.58e-04 (-2.63e-04, -7.32e-05) | <0.001 |
|  | Total Effect | -1.60e-04 (-2.64e-04, -7.48e-05) | <0.001 |
|  | Prop. Mediated | 0.94% (0.29%, 2.21%) | <0.001 |

HLCD, healthy low-carbohydrate diet; AD, Alzheimer’s Disease; VD, Vascular Dementia; ACME, average causal mediation effect; ADE, average direct effect; CI, confidence interval. Mediation analysis was performed using the Quasi-Bayesian Monte Carlo method with 1000 simulations. All models were adjusted for age, sex, Townsend scores, ethnicity, education, PM2.5, smoking status, alcohol intake, BMI, physical activity, hypertension, diabetes.

Supplementary Table 5 Associations between the Healthy Low-Carbohydrate Diet score and risks of Alzheimer’s Disease and Vascular Dementia

| Outcome | Variable | Events/N | Model 1 |  | Model 2 |  | Model 3 |  |
| --- | --- | --- | --- | --- | --- | --- | --- | --- |
|  |  |  | HR (95% CI) | P | HR (95% CI) | P | HR (95% CI) | P |
| AD | Q1 | 183/39367 | 1.00 (Ref) | -- | 1.00 (Ref) | -- | 1.00 (Ref) | -- |
|  | Q2 | 145/39366 | 0.78 (0.63-0.97) | 0.029 | 0.79 (0.63-0.98) | 0.033 | 0.81 (0.65-1.00) | 0.052 |
|  | Q3 | 124/39366 | 0.67 (0.53-0.84) | <0.001 | 0.71 (0.56-0.89) | 0.003 | 0.72 (0.57-0.91) | 0.006 |
|  | Q4 | 106/39366 | 0.57 (0.45-0.73) | <0.001 | 0.64 (0.51-0.82) | <0.001 | 0.66 (0.52-0.84) | <0.001 |
|  | HLCD (per 1-unit) | | 0.96 (0.95-0.98) | <0.001 | 0.97 (0.95-0.98) | <0.001 | 0.97 (0.96-0.99) | <0.001 |
| VD | Q1 | 115/39367 | 1.00 (Ref) | -- | 1.00 (Ref) | -- | 1.00 (Ref) | -- |
|  | Q2 | 80/39366 | 0.68 (0.51-0.90) | 0.007 | 0.67 (0.50-0.89) | 0.006 | 0.68 (0.51-0.91) | 0.009 |
|  | Q3 | 69/39366 | 0.59 (0.44-0.80) | <0.001 | 0.63 (0.47-0.85) | 0.002 | 0.64 (0.47-0.86) | 0.004 |
|  | Q4 | 54/39366 | 0.46 (0.33-0.64) | <0.001 | 0.53 (0.38-0.73) | <0.001 | 0.53 (0.39-0.74) | <0.001 |
|  | HLCD (per 1-unit) | | 0.95 (0.93-0.97) | <0.001 | 0.95 (0.93-0.97) | <0.001 | 0.96 (0.94-0.98) | <0.001 |

AD, Alzheimer’s Disease; VD, Vascular Dementia; HR, hazard ratio; CI, confidence interval. Model was adjusted for age, sex, Townsend scores, ethnicity, education, PM2.5, smoking status, alcohol intake, BMI, physical activity, hypertension, diabetes and total energy intake.

Supplementary Table 6 Joint association of HLCD score and frailty with the risk of Alzheimer’s Disease and Vascular Dementia

| Category | Events/N | Model 1 |  | Model 2 |  | Model 3 |  |
| --- | --- | --- | --- | --- | --- | --- | --- |
|  |  | HR (95% CI) | P | HR (95% CI) | P | HR (95% CI) | P |
| AD |  |  |  |  |  |  |  |
| Categories of HLCD-FI |  |  |  |  |  |  |  |
| Low-frail | 116/17,095 | 1.00 (Ref) | -- | 1.00 (Ref) | -- | 1.00 (Ref) | -- |
| Low-prefrail | 81/18117 | 0.64 (0.49-0.86) | 0.002 | 0.73 (0.55-0.97) | 0.032 | 0.72 (0.54-0.96) | 0.024 |
| Low-nonfrail | 131/43521 | 0.42 (0.33-0.55) | <0.001 | 0.56 (0.44-0.72) | <0.001 | 0.55 (0.43-0.71) | <0.001 |
| High-frail | 67/16783 | 0.58 (0.43-0.78) | <0.001 | 0.63 (0.46-0.85) | 0.002 | 0.63 (0.47-0.86) | 0.003 |
| High-prefrail | 62/17827 | 0.50 (0.36-0.67) | <0.001 | 0.63 (0.46-0.86) | 0.003 | 0.63 (0.46-0.86) | 0.003 |
| High-nonfrail | 101/44122 | 0.32 (0.25-0.42) | <0.001 | 0.46 (0.35-0.60) | <0.001 | 0.46 (0.35-0.60) | <0.001 |
| VD |  |  |  |  |  |  |  |
| Categories of HLCD-FI |  |  |  |  |  |  |  |
| Low-frail | 77/17,095 | 1.00 (Ref) | -- | 1.00 (Ref) | -- | 1.00 (Ref) | -- |
| Low-prefrail | 49/18117 | 0.58 (0.41-0.83) | 0.003 | 0.69 (0.48-0.98) | 0.039 | 0.67 (0.47-0.96) | 0.030 |
| Low-nonfrail | 69/43521 | 0.33 (0.24-0.46) | <0.001 | 0.44 (0.32-0.62) | <0.001 | 0.49 (0.35-0.68) | <0.001 |
| High-frail | 58/16783 | 0.74 (0.53-1.05) | 0.090 | 0.81 (0.58-1.15) | 0.237 | 0.83 (0.59-1.16) | 0.273 |
| High-prefrail | 33/17827 | 0.40 (0.26-0.60) | <0.001 | 0.53 (0.35-0.79) | 0.002 | 0.52 (0.35-0.79) | 0.002 |
| High-nonfrail | 32/44122 | 0.15 (0.10-0.23) | <0.001 | 0.23 (0.15-0.34) | <0.001 | 0.25 (0.17-0.38) | <0.001 |

AD, Alzheimer’s Disease; VD, Vascular Dementia; FI, Frailty Index; HR, hazard ratio; CI, confidence interval. Model was adjusted for age, sex, Townsend scores, ethnicity, education, PM2.5, smoking status, alcohol intake, BMI, physical activity, hypertension, diabetes and total energy intake.

Supplementary Table 7 Joint association of HLCD score and frailty with the risk of AD and VD after excluding AD or VD cases that occurred within the first five years of follow-up (N=155,870).

| Category | Events/N | Model 1 |  | Model 2 |  | Model 3 |  |
| --- | --- | --- | --- | --- | --- | --- | --- |
|  |  | HR (95% CI) | P | HR (95% CI) | P | HR (95% CI) | P |
| AD |  |  |  |  |  |  |  |
| Categories of HLCD-FI |  |  |  |  |  |  |  |
| Low-frail | 106/16,789 | 1.00 (Ref) | -- | 1.00 (Ref) | -- | 1.00 (Ref) | -- |
| Low-prefrail | 81/17897 | 0.70 (0.53-0.94) | 0.018 | 0.80 (0.60-1.07) | 0.135 | 0.79 (0.59-1.05) | 0.110 |
| Low-nonfrail | 128/43165 | 0.45 (0.35-0.59) | <0.001 | 0.60 (0.46-0.77) | <0.001 | 0.60 (0.46-0.77) | <0.001 |
| High-frail | 63/16526 | 0.60 (0.44-0.81) | 0.001 | 0.64 (0.47-0.88) | 0.006 | 0.65 (0.48-0.89) | 0.007 |
| High-prefrail | 59/17667 | 0.52 (0.37-0.71) | <0.001 | 0.66 (0.48-0.90) | 0.010 | 0.65 (0.47-0.90) | 0.009 |
| High-nonfrail | 100/43826 | 0.35 (0.26-0.46) | <0.001 | 0.49 (0.37-0.65) | <0.001 | 0.50 (0.38-0.65) | <0.001 |
| VD |  |  |  |  |  |  |  |
| Categories of HLCD-FI |  |  |  |  |  |  |  |
| Low-frail | 67/16,789 | 1.00 (Ref) | -- | 1.00 (Ref) | -- | 1.00 (Ref) | -- |
| Low-prefrail | 44/17897 | 0.60 (0.41-0.88) | 0.008 | 0.71 (0.48-1.03) | 0.073 | 0.69 (0.47-1.02) | 0.061 |
| Low-nonfrail | 65/43165 | 0.36 (0.25-0.50) | <0.001 | 0.48 (0.34-0.68) | <0.001 | 0.53 (0.37-0.75) | <0.001 |
| High-frail | 53/16526 | 0.78 (0.54-1.12) | 0.176 | 0.85 (0.59-1.22) | 0.386 | 0.87 (0.60-1.25) | 0.444 |
| High-prefrail | 31/17667 | 0.43 (0.28-0.66) | <0.001 | 0.57 (0.37-0.87) | 0.009 | 0.57 (0.37-0.87) | 0.009 |
| High-nonfrail | 31/43826 | 0.17 (0.11-0.26) | <0.001 | 0.25 (0.16-0.39) | <0.001 | 0.28 (0.18-0.44) | <0.001 |

AD, Alzheimer’s Disease; VD, Vascular Dementia; FI, Frailty Index; HR, hazard ratio; CI, confidence interval. Model was adjusted for age, sex, Townsend scores, ethnicity, education, PM2.5, smoking status, alcohol intake, BMI, physical activity, hypertension and diabetes
